# Quantification of the Culture Stability of Stem Cell Fractions from Oral-Derived, Human Mesenchymal Stem Cell Preparations: A Significant Step toward the Clinical Translation of Cell Therapies

**DOI:** 10.3390/cells12232703

**Published:** 2023-11-25

**Authors:** Hitesh Chopra, Chen Cao, Celia Sommer, Alex Dahlkemper, James Sugai, James L. Sherley, Darnell Kaigler

**Affiliations:** 1 Kaigler Lab of Stem Cell Science and Tissue Regeneration, Department of Periodontics and Oral Medicine, School of Dentistry, University of Michigan, Ann Arbor, MI 48109, USA; choprah@umich.edu (H.C.); cao.chen05@gmail.com (C.C.); cmsommer@umich.edu (C.S.); abdahlkemper@gmail.com (A.D.); jsugai@umich.edu (J.S.); 2 Asymmetrex®, LLC, Boston, MA 02130, USA; jsherley@asymmetrex.com; 3 Department of Biomedical Engineering, University of Michigan, Ann Arbor, MI 48109, USA

**Keywords:** mesenchymal stem cells, kinetic stem cell counting, alveolar bone, stem cell therapy, cell biomanufacturing

## Abstract

A continuing limitation and major challenge in the development and utilization of predictable stem cell therapies (SCTs) is the determination of the optimal dosages of stem cells. Herein, we report the quantification of stem cell fractions (SCF) of human mesenchymal stem cell (MSC) preparations derived from oral tissues. A novel computational methodology, kinetic stem cell (KSC) counting, was used to quantify the SCF and specific cell culture kinetics of stem cells in oral alveolar bone-derived MSC (aBMSCs) from eight patients. These analyses established, for the first time, that the SCF within these heterogeneous, mixed-cell populations differs significantly among donors, ranging from 7% to 77% (ANOVA *p* < 0.0001). Both the initial SCF of aBMSC preparations and changes in the level of the SCF with serial culture over time showed a high degree of inter-donor variation. Hence, it was revealed that the stability of the SCF of human aBMSC preparations during serial cell culture shows inter-donor variation, with some patient preparations exhibiting sufficient stability to support the long-term net expansion of stem cells. These findings provide important insights for the clinical-scale expansion and biomanufacturing of MSCs, which can facilitate establishing more effective and predictable outcomes in clinical trials and treatments employing SCT.

## 1. Introduction

Stem cell therapy (SCT) is a promising field of medicine that uses stem cells to treat or cure various diseases and conditions. However, one of the major limitations to efficacious and predictable SCT is the optimal dosing of stem cells. Unlike pharmaceutical and biopharmaceutical medicine, in which the therapeutic agents can be accurately dosed for predictable and standardized therapy, SCT does not have established and validated methodologies for the accurate quantification of treatment stem cells. As a result, clinical outcomes of stem cell therapy clinical trials [1,2,3,4,5], as well as approved stem cell treatments, are very difficult to predict, compare, and reproduce [6,7,8,9].

Currently, the three minimal criteria to define human MSCs are (1) an adherence to plastic in culture; (2) the positive expression of CD105, CD73, and CD90 and a lack of expression of CD45, CD34, CD14 or CD11b, CD79α or CD19, and HLA-DR surface molecules; and (3) the ability to differentiate into osteoblasts, adipocytes, and chondroblasts in vitro [10]. Despite these criteria being used widely in the field, in the context of translational studies toward the development of cell therapies, the heterogeneous nature of different MSC-containing populations confounds the understanding of how they can be used. MSC-containing populations from diverse tissue sources (with alveolar bone being one example) are, in fact, heterogeneous mixtures of stem cells, committed progenitor cells, and committed cells [8,11,12,13,14]. Currently, fluorescence-activated cell sorting (FACS) is used to purify and quantify specific cell populations based on immunophenotypes detected using flow cytometry. However, a specific MSC marker that can be used to determine the stem cell fraction (SCF) within a particular heterogeneous MSC-containing population is lacking. As such, despite being phenotypically similar, different MSC-containing populations may behave differently, particularly in their growth kinetics, and they may differ in their specific fraction of MSCs as well. These differences undoubtedly also impact how they behave in a therapeutic context. 

Towards the goal of optimizing the study of MSCs and their therapeutic efficacy, we investigated, for the first time, the SCF and cell culture kinetics of oral-derived human alveolar bone MSC (aBMSC)-containing preparations from different patients. For these studies, we employed a recently described method called kinetic stem cell (KSC) counting. KSC counting is a computational simulation method that provides the routine, reproducible, and accurate determination of the SCF of heterogeneous organ and tissue cell populations [13,15,16]. Here, we report for the first time the use of KSC counting to determine SCFs in oral-derived aBMSC-containing preparations. Our analyses establish that the SCF of these tissue cell preparations can vary significantly among donors. The KSC counting method also revealed that, unlike previously described human tissue stem-cell-containing preparations [15,16], including MSC-containing preparations from other sources (e.g., bone marrow and adipose tissue) [13,16], the SCF of some donor aBMSC-containing populations is stable with serial cell culture. This newly discovered property may inform future work to establish more effective biomanufacturing processes for MSCs, as well as other tissue stem cell types, for use in SCTs.

## 2. Materials and Methods

### 2.1. Isolation of Human aBMSC-Containing Preparations

MSC-containing cell populations derived from oral alveolar bone tissue (aBMSCs) from 8 patients (Table 1) were isolated in accordance with the University of Michigan, School of Dentistry Institutional Review Board guidelines under an approved protocol (IRB# #HUM00034368).

For aBMSC strain derivation, alveolar bone specimens were obtained from patients undergoing routine oral surgical procedures as previously described [12]. Briefly, a 2 mm core of the alveolar bone was surgically excised, following which, approximately 0.5 cc of marrow aspirate was obtained (range, 0.1–1.5 cc). These alveolar bone marrow tissue samples were re-suspended in minimum essential medium alpha (MEMα; Gibco, Carlsbad, CA, USA) and centrifuged at 600 g for 10 min at room temperature. The supernatant was removed, and the cell pellet was re-suspended in complete culture medium [CCM: MEMα supplemented with 15% FBS along with 1% antibiotic antimycotic (Gibco), 1% L-glutamine (Gibco), and 1% L ascorbic acid 2-phosphate (Gibco)] followed by transferring to T-25 tissue culture flasks. The flasks were left undisturbed without medium change for 5 days in a 37 °C humidified tissue culture incubator with a 5% CO_2_ atmosphere. Non-adherent cells were removed after 5 days in culture, and the medium was changed every three days thereafter. Once adherent cells reached 70–80% confluence, the cells were harvested via trypsinization, expanded, and cryopreserved. All eight aBMSC strains were derived using this procedure during the period from 12 September 2019 to 1 October 2019. 

### 2.2. Characterization of Human aBMSC-Containing Preparations

The expanded cells were first evaluated for their stemness using flow cytometry as previously described [17]. Briefly, human aBMSCs in T150 flasks, at 60–70% confluency, were harvested via trypsinization, filtered with a 70 µm cell strainer (to yield single cell suspensions), and aliquoted into 5 mL tubes at a concentration of 0.5 × 10^6^ cells/mL. These cell suspensions were then incubated with a blocking solution containing an anti-CD16/CD32 antibody (Biolegend, San Diego, CA, USA) at 4 °C for 10 min, followed by washing. Following this, these cell suspensions were incubated with CD73 (BV421 #344008), CD90 (FITC, Biolegend #328107), and CD105 (PE, Biolegend#323205) antibodies and their corresponding isotype controls (BV421#400157, FITC#400119, and PE#400113; all from Biolegend) at 4 °C for 30 min. After washing, these cells were analyzed using a MoFlo flow cytometer (Beckman Coulter, Brea, CA, USA).

### 2.3. Serial Cell Culture

Following characterization, cryopreserved cell samples were thawed and expanded for one passage to begin serial cultures for KSC counting analyses. Adherent serial cell cultures of the eight aBMSC strains were performed in triplicate in 6-well cell culture plates with 2 mL of CCM at 37 °C in humidified incubators with a 5% CO_2_ atmosphere. Briefly, 1 × 10^5^ cells/well were used to start cultures. Serial cell culture was performed as described previously for KSC counting analyses of MSC-containing tissue cell preparations [13], with the counting of live cells and dead cells at each culture passage based on trypan blue dye-exclusion. Cell counting was performed manually according to well established methods using a hemocytometer slide. Serial cell culture of all eight aBMSC strains was conducted in CCM on the same passage schedule, which was a transfer of 1/20 of the total recovered cells every 72 h, within a three-hour range of variance. As required for KSC counting analyses, serial cell cultures were continued until no cells could be detected [13,15].

### 2.4. KSC Counting Analyses

The live-cell and dead-cell count data from triplicate serial cultures were used to calculate triplicate sets of respective cumulative population doubling (CPD) data and dead cell fraction data over the entire period of serial cell culture. As described, these data were input in the TORTOISE Test^®^ KSC counting software (version 2.0) [15] to derive the initial SCF of each derived aBMSC strain. Thereafter, the SCF half-life (SCFHL) of each aBMSC strain during subsequent serial cell culture was determined using the previously described RABBIT Count^®^ software (version 1.0) [15]. The reported mean SCF and SCFHL determinations are based on 10 independent computer simulations as described [13,15].

### 2.5. Statistical Analyses 

The statistical confidence of SCF determinations and their corresponding TORTOISE Test^®^ software (version 2.0) simulation quality scores (SQS) were evaluated using Student’s two-tailed *t*-test. One-way ANOVA was used to assess the significance of variation in the SCF among the eight aBMSC strains. These statistical analyses were performed with 2020 GraphPad Prism 9 for macOS software, version 9.0.0.

## 3. Results

### 3.1. Isolation, Characterization, and Serial Cell Culture of Human aBMSC Strains

**Figure 1:**
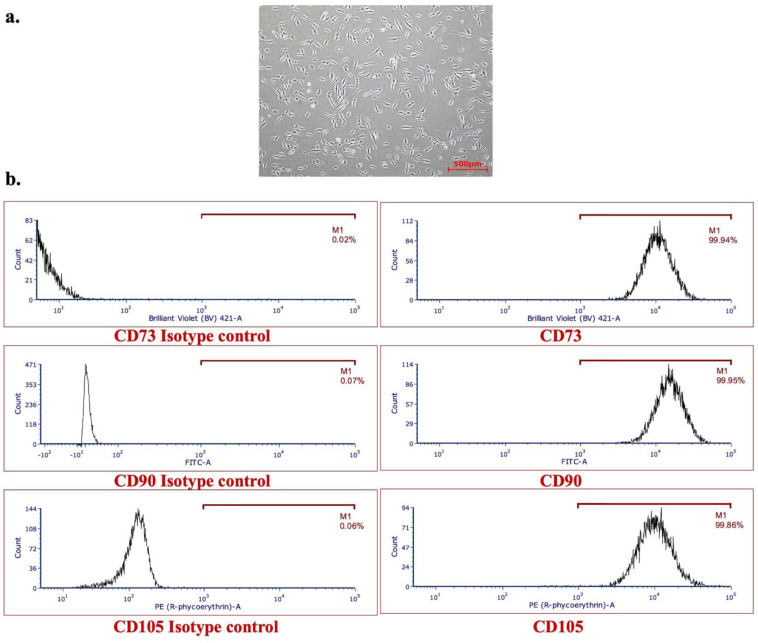
Isolation and characterization of aBMSC strains. (**a**) A representative image of an aBMSC strain in a complete culture medium after isolation from the alveolar bone. (**b**) Representative histograms of CD73, CD90, and CD105 along with their isotype controls obtained from analysis of flow cytometry data demonstrating expression of stem cell surface markers in aBMSC strain of P8.

Human aBMSC-containing preparations were successfully isolated from the alveolar bone marrow of eight patients, as we have previously described [12]. Table 1 provides the ages and sex of the patients. A representative image of an aBMSC cell strain in culture is shown in Figure 1a, as no qualitative difference was observed among all eight strains of aBMSC preparations with respect to their morphology.

Following expansion, the stemness of these aBMSCs strains was characterized using flow cytometry, which revealed that all strains expressed high levels (>95%) of expression of CD73+, CD90+, and CD105+ (Table 1, Figure 1b) surface markers.

**Figure 2:**
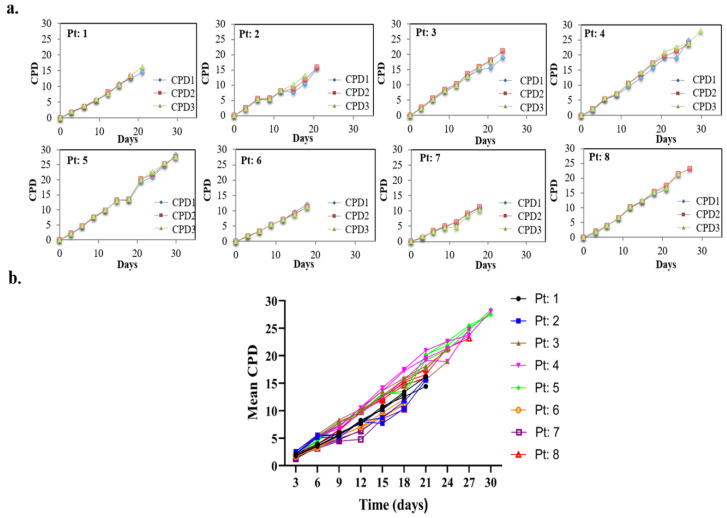
Cumulative population doubling (CPD) data from serial cell culture of the eight human aBMSC strains. (**a**) Graphs of the CPD data obtained for each of the eight aBMSC strains. The individual data for each of the parallel triplicate serial cell cultures (CPD1-CPD3) are plotted with respect to the day of cultures’ passage. (**b**) Comparison of the mean CPD data calculated from the triplicate serial cultures CPD data for each of the eight aBMSC strains.

Consecutively, each aBMSC strain was serially cultured as described above, beginning at the earliest passage (P2) after being thawed from cryopreservation. The required KSC counting serial cell culture endpoints of the aBMSC cultures, defined by reaching a point of no detectable cells, were achieved after 18 to 30 days (6–10 passages), with aBMSC strain from Patient 5 (Pt: 5) having a significantly higher number of passages (*p* < 0.05) in comparison to other cell populations, except Pt: 4 and Pt: 8. For all aBMSC strains, the variability among the CPD data for triplicate serial cell cultures was low (Figure 2a,b).

### 3.2. KSC Counting Analysis of the SCF of Human aBMSC Preparations

**Figure 3:**
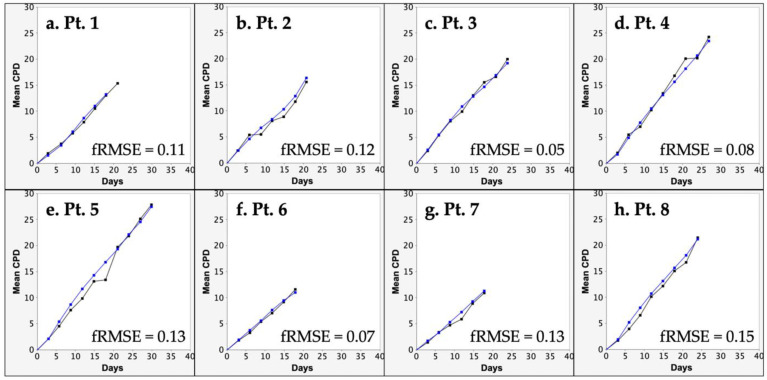
Evaluation of the quality of KSC counting simulation of the mean CPD data of human aBMSC strains. Shown are representative examples of KSC counting computer simulations of the mean CPD data reported in Figure 2. Black lines, mean CPD data. Blue lines, computer simulation. fRMSE indicates the quality of the depicted simulations’ approximation of the experimental mean CPD data.

From the input of replicate CPD data and mean dead-cell fraction data, the KSC counting TORTOISE Test^®^ software can be used to determine the mean SCF of primary tissue cell preparations [13,15,16]. The determination of SCF is achieved via the computational simulation of experimental replicate CPD data. The statistical confidence of SCF determinations depends on how well the experimental replicate CPD data are simulated. The quality of simulations is determined using a simulation quality score (SQS) [16]. As shown in Table 2, the mean SQS score based on 10 independent computer simulations was significantly less than the ideal low score of SQS ≤ 0.5 [16]. A form of representation of the quality of the computational simulations is shown in Figure 3. The root mean squared error/maximum mean CPD value (fRMSE) value in Figure 3 is a different metric than the SQS reported in Table 2. However, it is a related single graphical indicator of the overall SQS score. The fRMSE metric applies only to the quality of the instant simulation shown, whereas the SQS is an overall quality score for the computational simulation of the complete triplicate CPD datasets and their variance [16].

Table 2 lists the mean SCF values determined for the eight aBMSC preparations at P2 (see Materials and Methods) before subsequent cell culture. The values ranged from a low of 7% to a high of 77%. While some patient donor preparations had statistically equivalent SCFs (e.g., Patients 2, 3, 5, 6, and 7), others had significantly lower (Patient 1) or higher SCFs (Patients 4 and 8). The p-value for a single factor ANOVA of the SCF data was <0.0001, indicating highly significant inter-donor variation.

### 3.3. KSC Counting Analysis of the SCF Half-Life of Human aBMSC Preparations during Serial Cell Culture

We evaluated the stability of MSCs in aBMSC preparations during serial cell culture by using KSC counting to determine SCFHL, which measures how many CPD are required for a 50% decline in the SCF to occur. In addition to providing the initial SCF of serially cultured primary tissue cell preparations, KSC counting can be used to determine the SCF during subsequent periods of culture [16]. The previously described KSC counting RABBIT Count^®^ software can be used to relate the experimental mean CPD data of serial cell cultures to their corresponding values of SCF. For the many types of human tissue cell cultures examined to date, these data behave according to simple first-order decay kinetics [13,15]. Such SCF decay analyses can be used to determine the SCFHL of primary tissue cell cultures.

**Figure 4:**
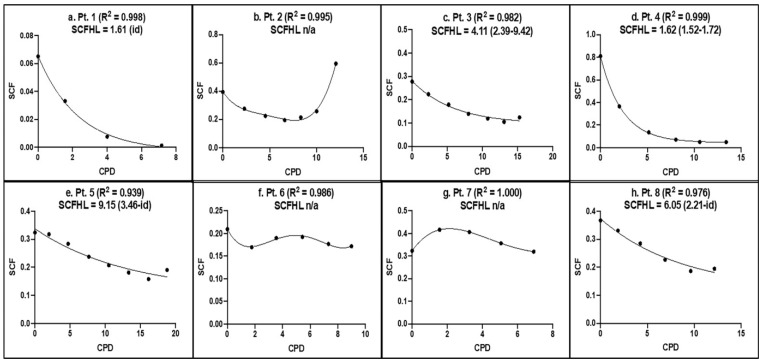
KSC counting analysis of the SCF half-life of patient-derived aBMSC strains during serial cell culture. The mean SCF determinations during serial cell culture of each aBMSC strain were plotted versus the respective experimentally determined mean CPD data. The plotted data were fitted to a simple first-order decay curve to estimate the SCFHL in CPDs. R2, the Pearson correlation coefficient for the quality of the fit of the data to first-order decay. ( ), 95% confidence interval. id, indeterminate or indeterminate interval boundary. n/a, not applicable.

Figure 4 shows the SCF versus CPD data and the SCFHLs determined for each aBMSC strain. Interestingly, three aBMSC strains did not show the characteristic decline in SCF with serial cell culture, as has been shown in SCF from other cell preparations [16]. Strains derived from Patients 2, 6, and 7 maintained the same level of SCF throughout, whereas in other Patients, SCFHL showed a more characteristic decline ranging from 1.61 CPD to 9.15 CPD. As in the case of SCF values, the aBMSC strains derived from some patients had equivalent SCFHLs (e.g., Patients 1 and 4), whereas others differed significantly (e.g., compare Patient 4 to Patients 5 and 8). Overall, the data indicate significant inter-donor variation in the SCFHL, a measure of the stability of MSCs during serial cell culture.

## 4. Discussion

Over the last two decades, there have been many preclinical and clinical regenerative studies employing the use of MSC-containing populations, with many of them oral-derived stem cells [18,19,20,21,22,23,24,25,26,27,28,29,30,31]. Despite similarities in the immunophenotype amongst different MSC-containing populations, it is not surprising that these studies have highly variable experimental outcomes due to the heterogeneous nature of MSC-containing populations. Currently, there is no consensus MSC marker that has been identified with which one can prospectively identify a “true MSC” in a mixed cell population. Without identifying specific MSC markers, it is impossible to quantify the actual number of stem cells within a given MSC-containing population. To our knowledge, we now report for the first time the accurate SCF in different oral-derived MSC-containing tissue cell populations.

The objectives of our study were to describe an approach that could enhance the predictability and standardization of cell therapies and decrease the potential for pre-clinical and clinical studies yielding contradictory outcomes. To this end, we attempted a precise determination of the proportion of “true” stem cells within these mixed populations. Although it is widely acknowledged that primary human cell preparations are heterogeneous [32,33,34], to date, there have been only a few characterizations of the SCF within these cell preparations [13,15,16]. Undoubtedly, in many cases, it may be differences in the SCFs of these cell populations that lead to differences in the reported outcomes of their use in different experimental contexts and certainly in different clinical contexts. 

The computational simulation method used to determine SCFs in this study is based on innate tissue cell kinetics that continues in ex vivo cell culture and has recently been described [15,16]. The method, KSC counting, is based on how tissue-specific stem cells divide asymmetrically to renew themselves while simultaneously producing non-stem committed progenitor cells, which progress to terminally-arrested cells [35,36]. KSC counting enables the ability not only to define the initial SCF of heterogeneous tissue cell preparations, but it also enables the monitoring of the SCF during serial cell culture [13,15,16]. This novel methodology has been validated for its specificity, accuracy, and reliability with respect to the severe combined immunodeficient (SCID) mouse repopulating cell assay, the only other method available for determining the specific fraction of a human tissue stem cell type so far [15,37,38].

In the present study, aBMSC preparations were isolated from eight patients and subjected to the KSC counting methodology to quantify and evaluate the SCF of the resulting cell strains during passage in culture. The initial CPD data analyses showed that the eight aBMSC strains had distinctive cell proliferation kinetics. The observed variation in the CPD kinetics of the different strains was not explained by general experimental variability because, in marked contrast, the triplicate CPD for each aBMSC strain showed a high degree of precision. The subsequent KSC counting analyses showed that these differences in CPD kinetics translated into a corresponding significant degree of inter-donor variation in the SCFs of the aBMSC preparations. To the authors’ knowledge, this finding constitutes the first demonstration of inter-donor variation in the SCF of tissue cell preparations isolated using the same procedures.

Although the same isolation procedures were used to prepare the eight individual aBMSC cell strains, we cannot exclude the possibility that the presently observed differences in SCF are due to variability in the performance of the isolation procedures, though we think this is an unlikely cause. The evaluations of SCF correlations with age or sex did not detect any significant patterns of association. However, this initial small-scale study of different donors lacks sufficient statistical power for a confident investigation of associations that might inform us about biological determinants of the observed inter-donor SCF variance.

A high degree of inter-donor variation was also detected for the SCF stability of aBMSC strains during serial cell culture. Of particular interest are three aBMSC strains (Patients 2, 6, and 7) that show a stable SCF throughout serial cell culture. Such stability predicts the net expansion of the MSCs in these preparations when they are cultured. This unusual feature was reported recently for human adipose-derived MSCs when cultured in a medium containing fetal bovine serum, in comparison with a medium containing a proprietary growth factor supplement used as a substitute for FBS [13]. The present study also used FBS as the medium supplement, although from different lots of FBS, which may underscore the influence of lot–lot variability of FBS and its effects on the serial culture of MSCs. 

To different degrees, the other aBMSC strains had SCFHLs indicative of the characteristic decline in SCF with culture [13,15,16]. The SCF of cultured tissue cells results from a complex interplay of the production kinetics and loss kinetics of the stem cells, transiently amplifying committed progenitor cells, and terminally arrested cells that compose the cell heterogeneity of primary tissue cell cultures [13,15,16]. A detailed KSC counting analyses of the predicted roles of these factors in determining the SCFHL of the aBMSC strains is currently ongoing and will be reported at a later time.

Unlike the SCF of the initial uncultured aBMSC preparations, the inter-donor variation in SCFHL cannot be readily attributed to possible variability in the isolation procedures. Given the similar serial cell culture conditions and procedures, the observed differences in SCFHL are very likely to manifest biological or clinical differences in the patient donors. However, no significant associations were detected between SCFHL and patient age or sex, and there was also no significant association detected between SCFHL and SCF. Further, as indicated for the SCF analyses, this initial small study lacks sufficient statistical power for a confident investigation of associations that might inform us about the biological determinants of the observed inter-donor SCFHL variance. 

Future follow-up studies should aim to not only increase patient samples in order to increase statistical power for further investigations of SCFs amongst different MSC samples from alveolar bone, but should also incorporate patient samples from different age groups (young, middle, and old age) and from different sources of MSCs. MSCs derived from other dental tissues, such as dental pulp (DPSCs), gingiva (GMSCs), and periodontal ligament (PDLSCs), or MSCs derived from other non-dental tissues, such as those from bone marrow (BMSCs), adipose, and muscle tissue, could have very different growth kinetics than those of aBMSCs. Finally, additional studies should consider the overall health status of patients to determine if systemic variables, such as systemic disease or environmental influences (i.e., smoking status), affect SCF. These comparative studies determining SCF differences between MSCs derived from various tissue sources (dental tissues or non-dental tissues), between different age groups, or between patients of different health status can provide valuable insights into not only the SCF, but also its stability and variability across different variables. In addition, preclinical investigations into how different types of MSC preparations and their subpopulations can synergistically influence SCF, stability, or therapeutic outcome can be elucidated. All such developments could accurately provide a predictable and reproducible means for stem cell dosing by ensuring a sufficient and consistent supply of MSCs when evaluated in a therapeutic context. This would, in turn, ameliorate the effectiveness of MSC biomanufacturing for stem-cell-based clinical trials, and significantly accelerate progress in the development of SCTs.

Finally, the limitations of the present study were, but not limited to, the relatively small sample size of MSC preparations which were analyzed, the time-sensitive nature of serial culturing, the inherent biases innate in manual cell counting, and the statistical variation in the KSC counting computational simulation. The continued development and optimization of standardized operating procedures to address these limitations will be instrumental in expanding on these findings in future studies.

## 5. Conclusions

In conclusion, the present study demonstrates, for the first time, the inter-donor variation in the SCF of a specific type of human tissue cell preparation. We propose that the inter-donor variation in SCFs defined herein for human aBMSC strains is likely to be a characteristic of SCFs of tissue cell preparations from other human organs and tissues, as well as from other vertebrate species. We also found that the subsequent serial culture of aBMSC preparations occurred with donor-specific variation in the stability of their SCF. Of particular interest, some aBMSC strains had stable SCFs, indicative of the ability to achieve net expansion of the MSCs in culture. These findings provide important insights for the clinical-scale expansion and biomanufacturing of MSCs, which can facilitate establishing more effective and predictable outcomes in clinical therapies employing MSC-based stem cell therapies.

## Figures and Tables

**Table 1:** Demographic data and quantitative expression (in %) of cell surface markers for alveolar bone-derived MSC (aBMSC) strain donor patients (P).

P	Sex	Age	CD73	CD90	CD105
1	F	90	99.66	98.39	98.96
2	M	56	99.81	99.86	99.38
3	F	78	99.92	99.80	99.29
4	F	62	99.77	99.80	99.10
5	M	61	99.62	99.84	99.80
6	F	49	99.92	99.94	99.91
7	F	25	99.54	99.65	99.73
8	M	43	99.94	99.95	99.86

**Table 2:** KSC counting mean simulation quality scores (SQS) and mean stem cell fractions (SCF) of eight human aBMSC strains.

Patient	Simulation Quality Score (SQS)			Stem Cell Fraction (SCF)		
	Mean	*p*-Value	95% CI	Mean ± SD	*p*-Value	95% CI
**1**	0.07	<0.0001	0.05–0.09	0.07 ± 0.02	<0.0001	0.05–0.08
**2**	0.18	<0.0001	0.13–0.22	0.30 ± 0.22	0.0018	0.15–0.46
**3**	0.07	<0.0001	0.05–0.09	0.26 ± 0.13	0.0001	0.17–0.35
**4**	0.13	<0.0001	0.12–0.13	0.77 ± 0.26	<0.0001	0.58–0.95
**5**	0.46	<0.0001	0.43–0.49	0.37 ± 0.15	<0.0001	0.26–0.48
**6**	0.22	<0.0001	0.20–0.24	0.22 ± 0.10	<0.0001	0.15–0.29
**7**	0.09	<0.0001	0.08–0.09	0.30 ± 0.16	0.0002	0.18–0.41
**8**	0.03	<0.0001	0.02–0.04	0.56 ± 0.18	<0.0001	0.43–0.69

## Data Availability

The data used to support the findings of this study are included in the article, whereas the supplemental data, which were not shown in the current study, can be made available from the corresponding authors on reasonable request.
